# Fresh-Air Cures in Towns

**Published:** 1905-10-21

**Authors:** Ernest L. Walford


### Editor's Letter-Box.

### FRESH-AIR CURES IN TOWNS.

*To the Editor of* The Hospital.

Sir,—As a constant reader of The Hospital, I saw the article in the number of the 9th inst. on "Fresh-air Cures in Towns." I have thought for a long time that it would be a great boon to many people in delicate health, and would prolong many lives, if the London County Council were to erect in some of the parks and on Hampstead Heath and Blackheath glazed colonnades open on one side to the air where people could sit in wet weather and get the benefit of the fresh air. Many a poor invalid could sit there and read or work and be restored to health.

Yours sincerely,
Ernest L. Walford.

